# Injury alters sensory, motor, and integrative elements underlying operant conditioning in the medicinal leech

**DOI:** 10.1371/journal.pone.0326039

**Published:** 2025-06-12

**Authors:** Ella R. Dockendorf, Brian D. Burrell

**Affiliations:** Division of Basic Biomedical Sciences, Sanford School of Medicine, Center for Brain and Behavior Research (CBBRe), University of South Dakota, Vermillion, South Dakota; Universidad de Guadalajara, MEXICO

## Abstract

Studies of pain/nociception often rely on simple reflexes to assess pain-related changes in behavior. However, there is considerable interest in utilizing more complex, self-initiated behaviors in place of stimulus-evoked reflexes. In this study we report an operant conditioning assay using *Hirudo verbana* (the medicinal leech) to assess the effects of injury on motivational and cognitive processes. Animals were placed in an arena consisting of an illuminated and a dark chamber with a connecting section in between. The connecting section was partially filled with gravel, which acted as an obstacle and delayed escape from the illuminated to the dark side. With repeated experience *H. verbana* learned to overcome the gravel obstacle, reflected as a decreased escape latency from the illuminated chamber. The capacity for this enhanced escape behavior was retained for up to two hours. In animals that received an injury to the posterior sucker, learning and memory of this operant escape task was disrupted. Injured animals also exhibited mechanosensory sensitization, changes in locomotion, changes in exploratory behavior, and increased negative phototaxis. Over 12 days, changes in locomotion, exploratory behavior, and phototaxis recovered to pre-injury levels, although mechanosensory sensitization remained. Disruptions in cognitive behavior also recovered during this period with the capacity for operant conditioning returning six days after injury and two hour retention of conditioning returning by day 12. This study shows that injury produces a complex and coordinated set of sensory, motor, and integrative changes in *H. verbana* that may be relevant to understanding the biological processes behind pain in vertebrates.

## Introduction

Pain is defined as “an unpleasant sensory and emotional experience associated with …actual or potential tissue damage” [1]. The sensory component of pain, nociception, involves the detection of damaging or potentially damaging stimuli from the periphery and transmitting it to the central nervous system (CNS). The emotional component of pain is more complex, encoding the aversive quality of damage-indicating stimuli that contributes to motivational, cognitive, and in some cases social behaviors that have a protective function [2]. Although it is convenient to delineate between the sensory and affective components of pain, interactions between the neural circuits that mediate these two elements are critical for the perception of painful stimuli and modulation of pain-elicited behaviors.

An animal’s response to pain has critical adaptive importance and failure to change behavior appropriately could ultimately lead to further damage or even death [3–5]. Even pain that can be characterized as chronic may have adaptive value [6]. That said, chronic pain conditions in humans, which can have a variety of etiologies, are a major health concern world-wide with many not receiving adequate relief from current, mostly pharmacological, therapies [7–11]. Consequently, there is a considerable need to understand the basic biology of pain. Studying pain in humans has obvious ethical constraints, but there are also limitations in how to quantitatively assess pain. Pain in humans is most often assessed through questionnaires, e.g., the Visual Analog Scale (VAS) or the Brief Pain Inventory, which have internal validity but are still self-reported, subjective measures [12–14]. The lack of biomarkers for pain in humans is addressed by the use of animal models to study pain, most often rodents. In these studies, increases or decreases in pain are often assessed via changes in stimulus-evoked responses to mechanical or thermal stimuli [15]. There are also a variety of experimental approaches to elicit persistent pain states in rodents, e.g., injection of inflammatory agents or damage to the peripheral or central nervous system [12]. The fact that known analgesics are also effective in these rodent preclinical models supports the validity of this approach [12]. However, findings based on stimulus evoked behaviors have had a poor track record of translating to effective clinical therapies due, at least in part, to their emphasis on the nociceptive sensory elements that detect damage and activate reflexive motor responses [12,16,17]. This has led to increasing efforts to utilize more complex, self-initiated behaviors as a way to assess pain states in animal models, e.g., learning to avoid aversive conditions that may increase pain, learning to prefer conditions that reduce pain, or changing the balance of time spent exposed to potential injury/death *versus* time in relative safety [4,12,18]. These more complex behaviors involve a combination of neural circuits mediating cognitive, motivational, and attentional processes, emphasizing the affective components of pain and therefore more representative of the human pain experience.

Given the evolutionarily conserved role of pain in adaptive fitness, there is value in comparative studies of nociception and pain in a diverse set of species across the animal kingdom. Therapies based on studies from one or a narrow range of species may not translate into effective treatments for humans because the physiological processes involved were more species-specific than appreciated. Comparative studies across vertebrate and invertebrate phyla, however, are more likely to uncover shared physiological processes mediating pain and nociception, leading to more effective therapies. There is considerable evidence of evolutionary conservation of the physiological mechanisms mediating pain and nociception between vertebrates and invertebrates, e.g., hyperactivity of primary nociceptors, phospholipid modulation of responses to noxious heat and chemical stimuli, and endocannabinoid modulation of synapses [19–24]. Invertebrate studies of pain and nociception have almost exclusively involved stimulus-evoked reflexive behaviors (notable exceptions include [5,25], but as with rodent studies, there is a need for assays testing more complex self-initiated behaviors in invertebrates.

The medicinal leech, *Hirudo verbana*, is an example of an invertebrate model organism that can contribute to understanding the basic biology of pain [20]. In terms of primary afferents, the leech nervous system has non-nociceptive rapidly-adapting (touch cells) and slow-adapting (pressure cells) mechanosensory neurons that are functionally comparable to slow- and fast-adapting Aβ fibers in mammals [26–28]. The leech also has distinct mechanical and polymodal nociceptors, the latter responding to strong mechanical force, high temperature, low pH, and noxious chemicals such as capsaicin and mustard oil [28–32]. Genes associated with mechanosensation in mammals are also expressed in *H. verbana* somatosensory neurons, e.g., piezo, DeG/ENaC, and trp [33]. Furthermore, there is detailed knowledge of the synaptic contacts between these afferents and various motor neurons, interneurons, and aminergic neurons allowing for direct, two-electrode recordings of identifiable synapses [34–38]. In many cases, leech nociceptive and non-nociceptive afferents converge on shared targets in the central nervous system, a feature that is also observed in mammals [39]. Disinhibition, an important mechanism in mammals for gating afferent input to nociceptive circuits, is also observed in *H. verbana* [40–44]. There has also been considerable research into sensitization of withdrawal reflexes as a result of nociceptive stimuli that have illustrated a role of serotonin which modulates pain in mammals as well [45–50]. Finally, the leech has been useful for studies of how the endocannabinoid system modulates nociception from the synaptic to the behavioral level [22]. The main endocannabinoid transmitters, 2-arachydonoylglycerol and N-arachidonoylethanolamide, are present of in *H. verbana* as are enzymes involved in endocannabinoid metabolism [51–53]. Both pro- and anti-nociceptive effects of endocannabinoids have been documented in the leech from the synaptic to the behavioral level [43,44,49,54–58], a feature that is also conserved in mammals [59–63].

**Fig 1:**
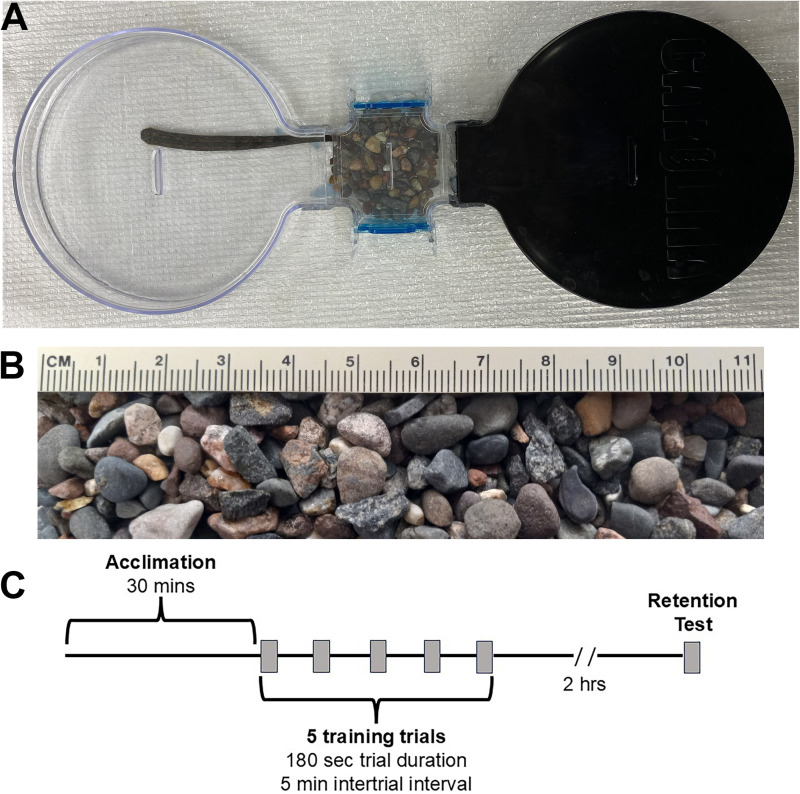
Illustration of the experimental setup, gravel used as mechanical obstacle, and experimental protocol. (A) The training arena consists of an illuminated chamber (left), a dark chamber (right), and a connecting section containing the gravel obstacle. A single leech can be seen with its posterior sucker anchored in the middle of the illuminated chamber and the anterior end extended to be in contact with the gravel in the connecting section. (B) High magnification view of gravel. (C) Experimental protocol. Following a 30 min acclimation period, a single leech was transferred to the illuminated chamber of the arena for a series of five trials interspersed with 5 minutes in the resting jar. Following the five trials, the animals were placed in the resting jar until undergoing the 2 hr retention test.

To date *H. verbana* studies of acute (minutes-hours) and persistent (days to weeks) injury-induced sensitization have relied on stimulus-evoked withdrawal reflexes [42,49,56,57]. Consequently there is a need to develop assays that involve more complex, self-initiated behaviors in the leech, e.g., a recent report of injury enhancing expression of pre-emptive evasion behaviors [64]. Here we examine operant conditioning in *H. verbana* and the effects of injury on such conditioning. Animals were placed in an arena consisting of a dark chamber, an illuminated chamber, and a connecting section between the two (Fig 1A). The connecting section is partially filled with gravel creating an obstacle to escape from the illuminated to the dark chamber. Since *H. verbana* are negatively phototaxic [65], they are motivated to overcome the gravel obstacle to escape from the illuminated chamber. We found that with repeated experience, escape latency from the illuminated side decreased with repeated trials in the arena and memory of this operantly conditioned behavior was retained for up to 2 hrs. In injured *H. verbana*, operant learning and memory was disrupted but appeared to recover over a 12-day period. This newly developed operant conditioning assay, therefore, provides a useful experimental approach in studying the effects of injury on motivational and cognitive behaviors using *H. verbana*.

## Methods

### Animals

Leeches (*Hirudo verbana*; 2–3 g) were obtained from a commercial supplier (North American BioPharma; Erie, CO). Animals were housed in plastic containers in artificial pond water (0.5 g sea salt per L of dH_2_O) that was changed every other day and housed in a refrigerated incubator maintained at 15°C with a 12 hr light/dark cycle. Animals were fed every six months with commercially available bovine blood (Animal Technologies, Tyler, TX or Hemostat Laboratories, Dixon, CA). Well-fed leeches can easily survive for up to 12 months from a single blood meal [66].

### Experimental Set-up

Behavioral experiments were carried out using commercially available large choice arenas from Carolina Biological Supply Company (catalog number 143051; Burlington, NC). The behavioral testing arena is shown in Fig 1A and consists of two large petri dishes or chambers (152 mm diameter, 25 mm deep) linked by a connecting section (70 mm long, 65 mm wide, 25 mm deep). One chamber was made of clear plastic and was illuminated by the room lights (740 lux) while the other chamber was made of opaque black plastic (referred to as the “illuminated” and “dark” chambers, respectively (Fig 1A). Each section of the arena had a lid to prevent the animal from escaping. The entire arena was filled with 300 mL pond water (depth = 10 mm). Gravel was used in the connecting bridge section as an aversive mechanical stimulus to impede escape from the illuminated to the dark chamber (Fig 1A and B; Kolorscape pea gravel, 4–12 mm in diameter). Gravel was chosen because it represented a more ethologically relevant stimulus that *H. verbana* might encounter in their aquatic environment. 0, 10, 20, or 40 g of gravel was evenly distributed across the length and width of the connecting chamber resulting in a uniform height of 10 g = 2 mm, 20 g = 4 mm, and 40 g = 8 mm. Leech behavior was video recorded (Logitec C920e webcam) at 30 frames/sec and digitally stored for playback when latency to escape the illuminated chamber and other behaviors were measured. The gravel was washed with deionized water prior to its first use in the chamber and was thoroughly washed between experiments. Between experiments, the Carolina chambers were washed with detergent and the gravel was washed with deionized water and then dried.

### Behavioral training procedures

Because leeches are housed in a refrigerated incubator at 15°C (see *Animals* sub-section) and are exothermic, all animals underwent a 30 min acclimation period in a closed jar filled with room temperature pond water. For all experiments, a trial started when a single leech was placed into the illuminated chamber of the arena. The trial ended when the leech reached the dark chamber or when the maximum trial period was reached.

Two sets of pilot experiments were carried out. The goal of the first set of experiments was to examine the effects of increasing amounts of gravel on escape latency. Escape latency was tested over five trials using 0, 10, 20, or 40 g of gravel in the connecting section with the trial duration of 5 mins and an intertrial interval of 5 mins when the leeches were maintained in their holding jars. The second set of pilot experiments was to test whether decreases in escape latency in the 40 g condition were retained 1 hr and 24 hrs following the last training trial. Here there were eight trials with a trial duration of 180 secs and an intertrial interval of 5 mins (again in holding jars). Trial duration was reduced to 180 secs because most animals escaped the illuminated chamber within this period in the 40 g condition during the first pilot experiment. Retention of this putative operant conditioning was tested at 1 and 24 hrs following the last trial. The retention tests consisted of reintroducing the leech to the illuminated chamber of the two-choice arena with 40 g in the connecting section (same conditions as during the training period). Escape latency was then re-tested over a 180 sec trial period. Animals that did not leave the illuminated chamber in this period were assigned an escape latency of 180 secs. Otherwise escape latency was based on when the leech entered the dark chamber (see S1 Video). In addition to escape latency, withdrawal from the gravel obstacle was also tested during the first pilot study. Withdrawal responses were counted in the 10 g and 40 g conditions to assess whether the gravel was aversive as a mechanosensory stimulus in addition to being a physical obstacle. Withdrawals were categorized as a rapid withdrawal of the anterior sucker from the gravel (see S2 Video) and were distinct from exploratory behavior (discussed below).

For all subsequent operant conditioning experiments, the training timeline is summarized in Fig 1C. The number of trials was reduced to 5 trials (180 sec duration, 5 min intertrial interval) with 40 g of gravel in the connecting section. Retention was tested at 2 hrs after the 5^th^ training trial. Testing retention at 2 hrs allowed time to interpose an extinction trial. For these extinction experiments, 1 hr after the 5^th^ conditioning trial, the leech was placed into a two-choice arena that had no gravel in the connecting section. One hour later (2 hrs after the 5^th^ training trial), the animal was placed back into the arena with 40 g of gravel in the connecting section (same conditions as during trials) and the escape latency measured over 180 secs.

For experiments testing the effect of injury, leeches were injured by piercing their posterior sucker with a T-pin which produces a persistent (3 weeks) sensitization to non-nociceptive mechanosensory stimuli [57]. After a 30 min rest period in individual jars, leeches were placed in the illuminated side of the arena and underwent the same five-trial training followed by a 2 hr retention test. Animals were then returned to their holding jars and placed in the refrigerated incubator until re-testing on Days 6 and 12. As with training on day 0, on days 6 and 12 animals underwent 30 min acclimation to room temperature, followed by operant conditioning, a 2 hr retention test. A subset of *H. verbana* were also tested for mechanosensitivity prior to injury and then following operant conditioning on post injury days 0, 6 and 12. Von Frey fibers were used to test the response threshold using the up-down method [57,67] to initiate the local shortening response. Local shortening is a defensive withdrawal reflex in which the muscle contraction is restricted to the stimulated segments and 1–2 neighboring segments [68].

### Assessment of locomotion

Two forms of locomotion were assessed, crawling and swimming. Crawling consists of an extension phase where the anterior sucker projects forward while the posterior sucker remains attached to the substrate and a retraction phase where the anterior sucker is now affixed to the substrate and the body contracts pulling the posterior sucker forward [69]. Examples of crawling can be seen in S1 Video. Swimming consists of S-shaped vertical undulations of the body traveling anterior to posterior [70] as seen in S3 Video. Time spent crawling or swimming during each training trial was measured in both injured and non-injured animals. One complication in measuring time crawling is that individual crawl steps could be interspersed with periods of no movement or exploratory behavior. To address this issue, whenever a single crawling step was not followed by a subsequent crawling step the time assigned to that step was set at 5 secs. This is based on the step duration reported in previous studies of crawling [69] that was also observed in this study. Because of this experimental constraint of capping the duration of a single step, a second measure of crawling was also used. Specifically, the number of crawl steps during each 180 sec trial was measured, which we refer to as crawling rate.

### Assessment of exploring

Exploring was defined as periods when the posterior sucker remained adhered to a substrate (dish wall, floor or lid) while the anterior sucker moved, but no crawling step was completed. These anterior movements were a combination of full body extensions, retractions, and bends, sometimes making contact with the physical objects in the arena, e.g., walls, floor, or gravel. Time spent exploring either the open arena or the gravel obstacle was assessed separately (see S4 and S5 Videos, respectively).

### Phototaxis experiments

*H. verbana* are negatively phototaxic, although they also use light and shadows as a means of detecting potential prey [65,71]. These animals possess simple eyes down the entire length of their body that are also radially distributed around the dorsal, ventral, and lateral body surfaces [72], allowing them to detect light omnidirectionally. To develop a more sensitive test of phototaxic behavior, a modified version of the two-choice arena was used in which both chambers were black/opaque with no gravel in the connecting section. A leech was placed in one chamber for 30 mins to acclimate and then the black lid of that chamber was replaced with a clear lid. In this way we created conditions in which light was only coming from one direction, i.e., from above. In this minimally illuminated chamber there is relatively little motivation for *H. verbana* to escape to the fully dark chamber. Negative phototaxis was tested prior to injury and then post-injury days 0, 6, and 12, the same time points that operant conditioning was tested after injury. Each leech was tested over a single 180 sec trial to cross from the clear-topped, minimally illuminated chamber to the completely dark chamber. Animals that did not escape to the completely dark chamber were assigned an escape latency of 180 sec.

### Statistical analysis

All statistics were carried out using GraphPad. 1-way or 2-way analysis of variance (ANOVA) tests or paired t-tests were carried out to test experimental effects of normally distributed data. In the case of ANOVAs, Tukey’s post-hoc test for multiple comparisons was used between experimental groups. In cases where only two sets of data were being compared, paired t-tests were used. A p ≤ 0.05 was required for statistical significance and assays producing p > 0.05 were listed as “n.s.” (not significant). All data is presented as the mean ± the standard error.

## Results

### Evidence of operant conditioning

Initial experiments were conducted to assess whether the amount of gravel in the connecting section affected the latency to escape from the illuminated to the dark chamber. As shown in S1A Fig , escape latency did increase with greater amounts of gravel. Escape latency did not differ between the 0 g and 10 g conditions during all five trials and the escape latency did not change across trials (N = 6 for both; trial duration = 300 secs). The escape latency in the 20 g and 40 g conditions (N = 6 for both) was greater, at least initially, compared to the 0 and 10g groups. A subsequent 2-way ANOVA confirmed a significant effect of gravel amount on escape latency (F_3,100_ = 12.75, p < 0.0001). Post hoc analysis found no significant difference in escape latencies between the 0 and 10 g group. However, the 20 g group was significantly different from the 0 g (p < 0.005) and 10 g (p < 0.05) and the 40 g group was also significantly different from the 0 g (p < 0.0001) and 10 g (p < 0.0001) groups. Interestingly, escape latency in the 20 and 40 g conditions decreased over the five trials such that these groups had the same escape latencies as 0 and 10 g groups by trial 5. The 2-way ANOVA detected a significant trial effect (F_4,100_ = 4.53, p < 0.005), but no interaction effect (F_12,100_ = 1.84, ns). These findings indicate that 40 g of gravel in the connecting section is an effective obstacle that significantly delays *H. verbana* escape from the illuminated chamber. However, escape latency decreased with repeated exposures to the training environment, suggesting an experience-dependent process in which leeches learned that they could overcome the gravel obstacle, i.e., operant conditioning.

There are multiple reasons why 40 g of gravel acts as an obstacle for escaping the illuminated chamber. It is likely that the gravel is a physical barrier that is either difficult to climb over and/or obscures the leech’s visual perception of the dark chamber. As noted in the Methods, increasing amounts of gravel led to an increasingly higher barrier in the connecting chamber (2–8 mm). It is also possible that the gravel is a mechanically aversive substrate to *H. verbana*. Although gravel was chosen as a substrate that *H. verbana* would normally encounter in its aquatic environment, there is evidence that leeches have an aversion to rough textured surfaces [73]. We examined the recordings from these initial experiment to determine if there were differences in the initial encounters with the least amount of gravel (10 g) *versus* the greatest amount (40 g). *H. verbana* exhibited withdrawals from 40 g gravel in the connecting chamber that were not observed in the 10 g condition (S1B Fig). These withdrawals were distinct from exploratory interactions also observed with the gravel (see S2 and S5 Videos). A 2-way ANOVA comparing the number of withdrawals between the 10 g and 40 g groups confirmed statistically significant effects for the gravel amount (F_1,70_ = 58.33, p < 0.0001) and trial (F_4,70_ = 8.26, p < 0.0001), as well as an interaction effect (F_4,70_ = 6.51, p < 0.0005). From these initial experiments, we conclude that when given a choice *H. verbana* will quickly escape to the illuminated to the dark chamber, consistent with previous description of negative phototaxis in the leech [65]. Furthermore, increasing amounts of gravel impede initial escape from the illuminated chamber, but escape latency decreases with repeated trials, suggesting an operant learning process in which the leech learns that the gravel is an obstacle can be overcome.

### Retention and extinction of operant learning

A second set of pilot experiments was carried out to test for retention of this putative learned behavior. Using the same testing arena with 40 g of gravel in the connecting section, *H. verbana* underwent eight trials (180 sec trial duration) in the arena with a 5 min intertrial interval. The increased number of trials was from five to eight was to determine if more training produced further decreases in escape latency compared to what was observed in S1 Fig. The decrease in the trial duration from 300 secs to 180 secs was based on the observation that most escapes occurred within 180 secs. Escape latency was re-tested 1 hr and 24 hrs following the last training trial, again using the same testing arena with 40 g of gravel in the connecting section. If the escape latency at these two time points was still significantly reduced compared to trial 1, this would indicate retention of the learned escape behavior. Escape latency did significantly decrease over the eight trials (S2 Fig; 1-way ANOVA F = 6.22, p < 0.0001; N = 8), although additional trials did not produce any further reduction in latency. Consequently, all subsequent operant conditioning experiments used five training trials with a 180 sec trial duration. When behavior was re-tested at 1 and 24 hrs (S2 Fig), escape latency at 1 hr was still significantly reduced compared to trial 1 levels (paired t = 7.02, p < 0.001), indicating retention of the learned escape behavior. Escape latency increased at 24 hrs and was not different from trial 1 (t = 1.85, ns) indicating no retention over at this time point.

**Fig 2:**
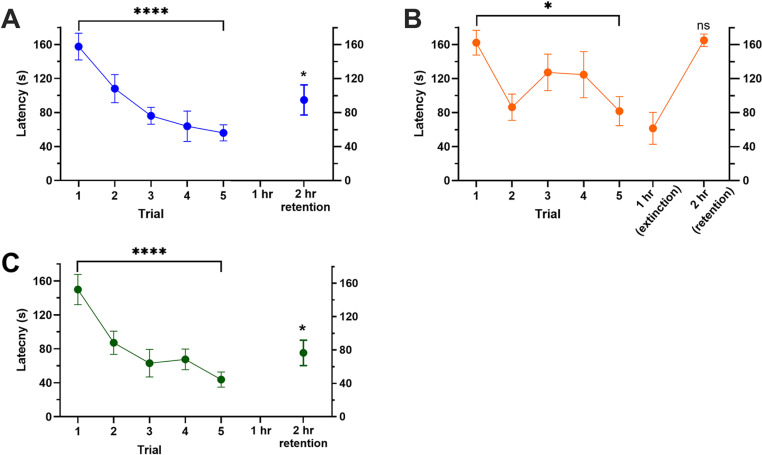
Retention and extinction of operant learning. (A) Following 5 training trials (180 secs duration, 5 min ITI), evidence of retention was observed 2 hrs after training. (B) Following 5 trial training trials, leeches were exposed to the arena without gravel in the connecting section 1 hr after training. No evidence of 2 hr retention was observed following this extinction trial. (C) Operant conditioning and 2 hr retention were still observed when animals were trained in a different chamber for each trial. *p < 0.05, ***p < 0.001, ns = not significant.

Next, we examined whether conditioned decreases in escape latency could be extinguished. In order to have post-training intervals where retention following training and retention following training+extinction could be compared, the post-training retention period was increased to two hours after the last (fifth) training trial (Fig 1C). This allowed for the placement of a single extinction trial one hour after the fifth training trial. As shown in Fig 2A a significant decrease was again observed in escape latency over the five trials (F_4,35_ = 8.34, p < 0.0001; N = 8). Furthermore, the escape latency during the 2 hr retention test was significantly reduced compared to trial 1 (t = 2.61, p < 0.05) indicating that the learned escape behavior was retained. To test whether this operant conditioning could be extinguished, training was repeated in a second group of animals with the addition of an exposure to the arena without the gravel obstacle 1 hr post-training (extinction trial duration = 180 secs). This was followed by a re-test of escape latency in the 40 g of gravel condition at the 2 hr time point. It was hypothesized that exposure to the arena without gravel obstacle following training would cause extinction of the operant escape memory, thus no enhanced escape latency would be observed during the 2 hr retention test. Once again, a significant decrease in escape latency over the five training trials was observed in this group (Fig 2B; F_4,35_ = 2.82, p < 0.05; N = 8). Response latency during the 1 hr extinction trial was also low but this was expected because there was no gravel obstacle in the connecting section at this time. In the subsequent 2 hr retention test with 40 g of gravel, the escape latency had increased and was not significantly different from trial 1 (Fig 2B; t = 0.26, ns), indicating no retention of the learned escape behavior. These findings are consistent with extinction of the conditioned operant escape behavior.

In previous studies of annelid learning using earthworms, operant conditioning in a T-maze was later found to actually be mediated by pheromones [74,75]. These pheromones could be aversive (alarm pheromones) to avoid the punishment side of the T-maze or attractive in nature to indicate the non-punished side. In the present study, the fact that extinction experiments were carried out in the same chamber as the training and retention tests would argue against pheromones that either signaled aversion to the illuminated chamber or attraction to the dark chamber. However, it is possible that *H. verbana* mark a path over the gravel obstacle leading to the dark chamber that could be followed in later training trials or during the retention test. To assess this possibility, a group of animals underwent the same training protocol (five training trials + 2 hr retention test), but each of the five training trials was conducted in a new arena so there was no opportunity for potential pheromone release to affect escape latency. If changes in escape behavior are due to pheromones, then one would expect to see no decrease escape latency using this protocol (i.e., using a new chamber every trial). A significant decrease in escape latency during training was still observed in this group (Fig 2C; F_4,35_ = 8.31, p < 0.0001; N = 8) that was indistinguishable from the results when an animal was trained in a single arena (Fig 2A). In addition, escape latency during the 2 hr retention test was significantly less than the trial 1 latency (Fig 2C; t = 2.93, p < 0.05), indicating that retention was also observed. These findings argue against the role of pheromones contributing to decreases in escape latency during training or subsequent retention.

### Effects of injury on operant conditioning

Next, we examined the effects of injury on operant conditioning in *H. verbana*. Animals were injured using a T-pin to pierce the posterior sucker (N = 14). This approach produces robust and long-lasting (≈ 3 weeks) sensitization of reflexive withdrawals to non-nociceptive mechanosensory stimuli and increases pre-emptive locomotion and evasion behaviors [57,64]. Injured animals underwent operant conditioning training followed by a 2 hr retention test (Fig 1C) on the same day of injury (day 0) and then 6 and 12 days following injury (see *Methods* for more details). Re-testing was carried out on these days to examine how injury affected operant learning well within the 3-week period that these animals are sensitized.

**Fig 3:**
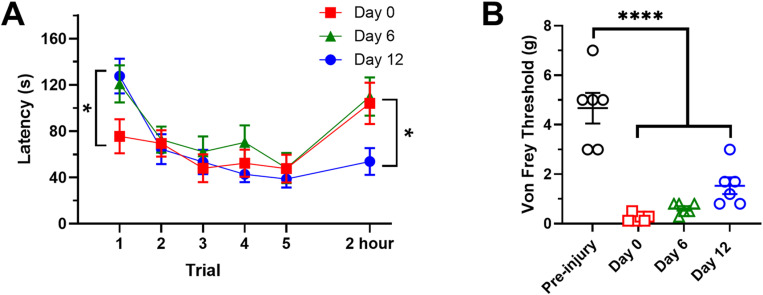
Effects of injury on operant conditioning and memory. (A) Animals undergoing operant conditioning on the same day as injury (Day 0, red) had a reduced escape latency at trial 1, showed no evidence of conditioning over the five training trials, and no evidence of retention of conditioning when measured 2 hrs later. On day 6 (green), trial 1 escape latencies were higher and there was evidence of conditioning over the five training trials, but 2 hr retention was still not observed. On day 12 (blue), both operant conditioning and 2 hr retention of the learned behavior was observed. * with left vertical bracket indicates a significant effect of injury day on trial 1 escape latency (p < 0.05). * with right vertical bracket indicates a significant effect of injury day on 2 hr retention (p < 0.05). (B) Response threshold to non-nociceptive mechanostimulation was significantly reduced compared to pre-injury levels immediately following injury (day 0) and remained reduced when measured on days 6 and 12. **** indicates post-hoc analysis at a level of p < 0.0001.

The effects of injury on behavior during operant conditioning and the 2 hr retention test were complex (Fig 3A). First, on day 0 the initial (i.e., trial 1) escape latency from the illuminated chamber was substantially lower compared to previous experiments using non-injured animals. This effect was only observed on day 0, with trial 1 latency being higher when these same injured animals were re-tested on days 6 and 12 (1-was ANOVA F_2,39_ = 3.43, p < 0.05). Second, the change in escape latencies over the five training trials also differed with post-injury day. Animals tested on day 0 exhibited no decrease on response latency over the five trials (1-way ANOVA F_4,65_ = 1.09, ns). However, decreases in escape latency across trials were observed when these leeches were tested on day 6 (F_4,65_ = 4.00, p < 0.01) and day 12 (F_4,65_ = 10.76, p < 0.001). Third, the escape latency during the 2 hr retention test was also influenced by the post-injury day. On days 0 and 6, escape latency during the 2 hr retention test actually increased, suggesting there was no memory of the operant conditioning. By day 12 the capacity to retain the operant conditioning had returned. A 1-way ANOVA confirmed an effect of injury on the escape latency measured during the 2 hr retention test (F_2,39_ trial = 3.92, p < 0.05).

Mechanosensory sensitivity was tested in a subset of the trained animals (N = 6) prior to injury, after injury on day 0 and again on days 6 and 12 using the up-down method [67] with manual von Frey fibers to stimulate the local shortening reflex. Local shortening is a withdrawal behavior consisting of contraction of the stimulated segment and 1–2 adjacent segments [68]. These tests were carried out to observe whether changes in mechanosensitivity occurred in parallel with changes in operant conditioning following injury. Consistent with previous experiments [57], this injury model elicited a significant decrease in the response threshold using von Frey fibers to stimulate the local shortening withdrawal reflex (Fig 3B) that was observed from Day 0 to Day 12 (1-way ANOVA F_3,20_ = 32.20, p < 0.0001). The level of von Frey mechanostimulation required to elicit the withdrawal reflex in both injured and non-injured animals was well below the threshold for activating mechanosensory nociceptors in *H. verbana* [26,30], so this change in response threshold represents a form of sensitization to non-nociceptive stimuli. Note that while operant learning and memory appeared to recover over the 12 day post-injury period, no recovery was observed in non-nociceptive mechanosensory sensitization.

**Fig 4:**
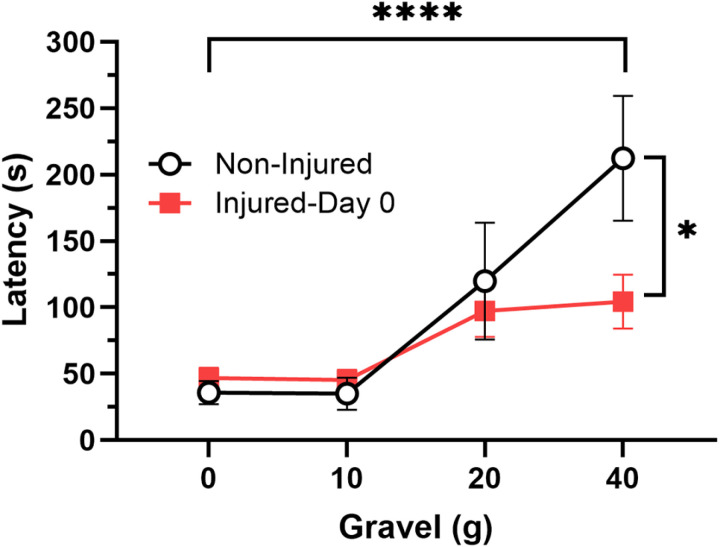
Effects of increasing gravel on initial escape latency in non-injured *vs.* **Day 0 injured leeches had similar initial escape latencies to non-injured animals at 0, 10, and 20 g of gravel.** However latency was significantly decrease for the 40 g condition in the injured day 0 animals. **** indicates significant differences across increasing amounts of gravel (p < 0.0001). * indicates a post-hoc test confirming a significant difference between injured and non-injured animals 40 g (p < 0.05).

The observation that injury appeared to reduce initial escape latency on day 0 prompted us to make a direct comparison of escape latencies in injured and non-injured animals with different amounts of gravel in the connecting section. To this end, escape latency was measured in non-injured (N = 6) and injured-day 0 (N = 8) animals in arenas with 0, 10, 20 and 40 g of gravel. As shown in Fig 4, escape latency with 0, 10, and 20 g of gravel was not affected by injury, but the latency to escape with 40 g of gravel in the connecting chamber was substantially reduced. This was confirmed by a 2-way ANOVA that showed a significant effect of increasing amount of gravel in both injured and uninjured animals (F_3,48_ = 11.87, p < 0.0001), no overall effect of injury (F_1,48_ = 2.68, ns), but a significant injury-gravel interaction effect (F_3,48_ = 2.84, p < 0.05). A post-hoc analysis showed that in chambers with 40 g of gravel there was a significant reduction in escape latency in the injured group compared to non-injured animals. These data are consistent with the reduced escape latencies observed in injured-day 0 leeches (Fig 3A) during trial 1 and confirm that injury enhanced escape from the illuminated chamber in spite of the gravel obstacle.

### Effects of injury on locomotion

**Fig 5:**
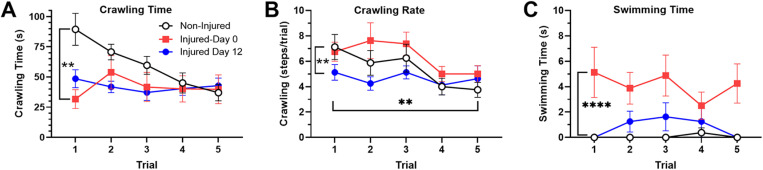
Effects of injury on locomotion during operant conditioning. (A) Initial time spent crawling in injured animals was reduced compared to non-injured animals. (B) When crawling rate was measured, injured-day 12 animals appeared to be slower compared to non-injured and injured-day 0 groups. (C) Injured-day 0 leeches spent more time engaged in swimming compared to the other two groups. (**p < 0.01, ****p < 0.0001).

To address whether enhanced escape behavior on day 0 is due to an increased locomotion, we first compared crawling during operant conditioning in non-injured, injured-day 0 and injured-day 12 animals. Crawling was the primary form of locomotion observed because the water depth is relatively shallow and not conducive to swimming (see S1 Video). Crawling time for non-injured *H. verbana* was initially high and decreased over the five-trial training period, reflecting the reduced time spent in the illuminated chamber due to operant conditioning (Fig 5A). In both injured-day 0 and injured-day 12 leeches, crawling time was reduced compared to non-injured animals during early trials (1–3) and did not decrease over five trials. A 2-way ANOVA confirmed the effect of injury on crawling time (F_2,105_ = 7.00, p < 0.01) and post-hoc analyses confirmed that crawling time in the injured-day 0 and -day 12 groups reduced compared to the non-injured group (p < 0.01 for both). Although no significant trial effect was observed (F_4,105_ = 2.15, ns), a significant injury-trial interaction effect was observed (F_8,105_ = 2.05, p < 0.05), consistent with the observation that crawling time did decrease in the non-injured animals over five trials, but not in either of the injured groups.

To address the possibility that injury may produce a change in the rate of crawling rather than time spent crawling, the number of crawling steps per trial (180 secs) were examined (Fig 5B). Here the results were somewhat different. A significant decrease in steps/trials across the five training trials was observed across all groups (2-way ANOVA, effect of trial F_4,105_ = 4.01, p < 0.05). Again, the decrease in steps/trial is likely a consequence of the reduced amount of time each animal spent in the illuminated chamber as a result of operant conditioning. A significant effect of injury was also observed (F_2,105_ = 5.13, p < 0.01) but here post hoc analysis indicated significant increase in crawling rate between the injured-day 0 and injured-day 12 groups (p < 0.01) but not between the non-injured and injured-day 0 leeches. No significant interaction effect was observed (F_8,105_ = 0.82, ns). It is unclear why the effects of injury on crawling time *versus* crawling rate differ. Nevertheless, injury-induced decreases in escape latency do not appear to be due to increases in crawling-type locomotion since injury either reduced amount or rate of crawling or have no effect (e.g., crawling rate in injured-day 0 group).

Interestingly, injury seemed to promote swimming behavior (Fig 5C; see S3 Video). This was surprising because the shallow water depth would seem to preclude swimming and swimming was almost entirely absent in non-injured animals. However, time swimming was significantly increased in injured-day 0 *H. verbana* compared to both non-injured and injured-Day 12 leeches. A 2-way ANOVA confirmed a significant effect of injury (F_2,105_ = 22.97, p < 0.0001), with post-hoc analysis showing significant differences in swimming time between the injured-day 0 group *versus* the other two groups (p < 0.0001 for both). No effect of trial (F_4,105_ = 0.30, ns) nor injury-trial interaction (F_8,105_ = 0.69, ns) was observed. The observed swimming in injured animals was not an uncontrolled response to the sucker injury since there was a 30 min period between the injury and the beginning of the behavioral training. Furthermore, while swimming did increase in injured animals, it is unclear whether swimming contributed to injury-induced enhancement of escape behavior. The time spent swimming in injured-day 0 leeches was only 2–4 secs per trial (Fig 5C), while the overall escape latency in these same animals was 40–80 secs (Fig 3A).

### Effects of injury on exploratory behavior

**Fig 6:**
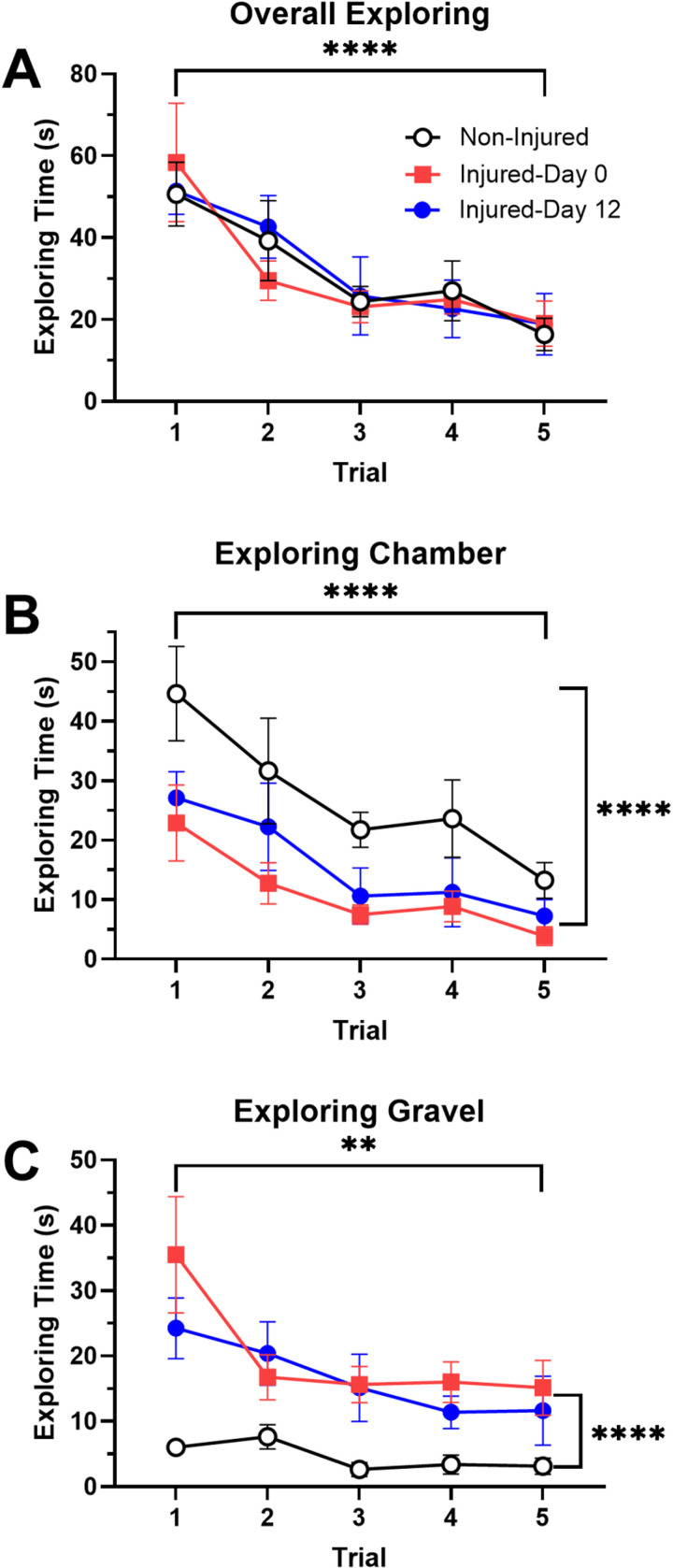
Effects of injury on exploratory behavior. (A) Injury did not affect total exploring time prior to exiting the illuminated chamber. (B) Non-injured animals engaged in more exploratory behavior of the illuminated chamber than did injured-day 0 or -day 12 animals. (C) Both injured-day 0 and -day 12 leeches spent more time exploring the gravel in the connecting chamber than did the non-injured animals. Vertical bracket indicate a significant difference between the injured and non-injured groups. Horizontal brackets indicate a significant effect of trial. **p < 0.01, ****p < 0.0001.

**Fig 7:**
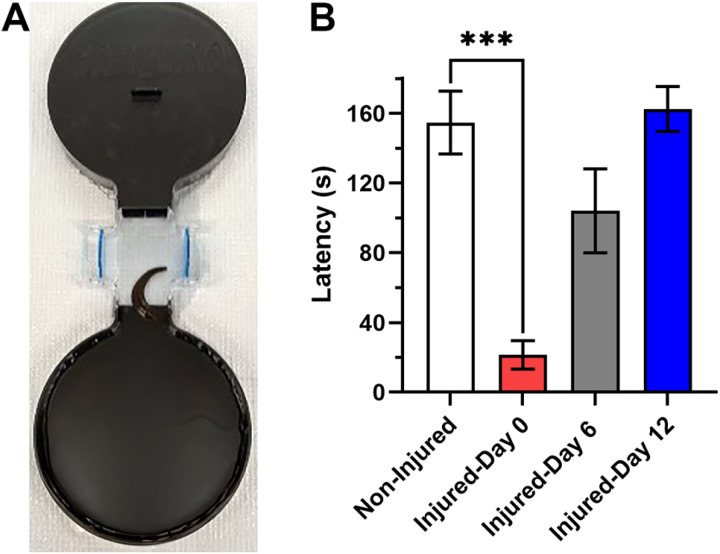
Effect of injury on negative phototaxis. (A) Two-choice arena used for phototaxis experiments. A leech can be seen transitioning from the chamber with a transparent lid towards the completely dark chamber. (B) Escape latencies measured on injured-day 0 were significantly reduced compared to non-injured animals.

Next, we examined the effects of injury on exploratory behavior. As shown in Fig 6A, there was no effect of injury on the total time spent exploring (F_2,105_ = 0.04, ns). Time spent exploring did decrease over the five training trials (F_4,105_ = 10.88, p < 0.0001), which again is an artifact of *H. verbana* across all groups spending less time in the illuminated chamber during the later trials. No injury-trial interaction effect was observed (F_1,70_ = 0.26, ns). Interestingly, when exploring behavior was divided into time spent exploring the surrounding illuminated chamber *versus* time spent exploring gravel in the connecting chamber (see S4 and S5 Videos), differences between non-injured and injured animals were observed. Injured-day 0 and -day 12 *H. verbana* spent significantly less time exploring the surrounding illuminated chamber compared to non-injured animals (Fig 7B; F_1,70_ = 12.33, p < 0.0001). A significant decrease in illuminated chamber exploring time over the five trials was also observed (F_4,70_ = 9.29, p < 0.0001), but there was no injury-trial interaction effect (F_1,70_ = 0.30, ns). The opposite pattern was observed in terms of time spent exploring gravel in the connecting chamber. Here injured-day 0 and -day 12 *H. verbana* spent significantly more time exploring the gravel compared to the non-injured animals (Fig 7C; F_1,70_ = 20.13, p < 0.0001). Again, a significant decrease in gravel exploring time over the five training trials was observed that appeared to apply across all three groups (F_4,70_ = 4.75, p < 0.01), but no injury-trial interaction effect was observed (F_1,70_ = 0.32, ns). In summary, injury significantly affected exploratory behavior in *H. verbana*, decreasing the amount of time spent exploring in the illuminated chamber, but increasing the amount of time exploring the gravel in the connecting chamber.

### Effects of injury on negative phototaxis

Since escaping the illuminated chamber was a critical element of this operant conditioning, the effects of injury on negative phototaxis were tested. *H. verbana* are negatively phototaxic, although they are also attracted to patterns of alternating light and dark indicative of surface waves produced by potential prey [65,71,76]. The *H. verbana* visual system is an array of simple eyes distributed on the anterior, ventral, and lateral body surfaces allowing them to detect light coming from any direction [72]. Because of this omnidirectional sensitivity to light, a different two-choice arena was used in which both chambers were completely opaque. After a 30 min acclimation period in one of these fully dark chambers the black lid was replaced with a clear lid allowing light to come from above but not from the sides and below (compare Fig 7A to Fig 1A). This has the advantage of more closely replicating the normal environment of *H. verbana* where most of the light is likely coming from above and not from the sides or below. Note, that no gravel was placed in the connecting section, therefore these conditions are most similar to the 0 g gravel group in S1A Fig. Each leech was tested over a single 180 sec trial to cross from the clear-top chamber to the completely dark chamber. Animals that did not escape to the completely dark chamber were assigned an escape latency of 180 sec. The escape latency under these conditioning was much higher when compared to non-injured animals tested in the standard arena with no gravel obstacle with a mean±SE of 155 ± 18 secs in the minimally illuminated arena *versus* 23 ± 5 secs in the standard area (compare Fig 7B and to the 0 g group in S1A Fig). This supports the idea that the level of photostimulation is reduced in these arenas with two dark chambers compared to our standard arenas.

When these animals were re-tested following injury (day 0), they exhibited considerably reduced escape latency, indicating enhanced negative phototaxis even under these minimally illuminated conditions (Fig 7B). Escape latencies increased when testing was repeated on day 6 and day 12 indicating a recovery of negative phototaxis to pre-injury levels. A 1-way ANOVA confirmed a significant effect of injury on escape latency (F_3,28_ = 14.78, p < 0.0001, N = 8 for all groups), with post-hoc analysis confirming that the injured-day 0 animals were significantly different compared to non-injured leeches (p < 0.0001), but there were no differences between the non-injured leeches and those tested on days 6 and 12. These results indicate that injury to the posterior sucker in *H. verbana* produced substantial sensitization of negative phototaxis.

## Discussion

From this study we show that injury imposes a complex set of behavioral changes in *H. verbana* that can be assessed using this operant conditioning assay. *H. verbana* have an initial resistance to crossing the gravel in the connecting section to escape the illuminated chamber. This is likely due to the 40 g of gravel being a physical obstacle that a leech has to climb over, a visual obstacle that impedes perception of the dark chamber, and the gravel being a mildly aversive mechanosensory stimulus. In a related species of aquatic leech there is a preference for smooth *versus* rough textured surfaces [73] and we observed withdrawals from the 40 g of gravel that were not observed in the 10 g gravel condition. With repeated exposures to the training arena, the animals learn that they can overcome the gravel obstacle as indicated by decreases in escape latency from the illuminated chamber. Memory of this operant conditioning following five training trials lasts for up to 2 hrs. Extinction of this 2 hr operant conditioning memory was observed when the animals were placed in a two-choice arena with no gravel obstacle and then returned to the 40 g gravel condition. There was a possibility that decreases in escape latency were due to pheromones released by *H. verbana* that guided escape into the dark chamber based on previous studies using earthworms, which are also annelids. Initial reports stated that worms avoided entering a T-maze arm in which they would receive an electric shock due to operant conditioning, but it was subsequently found that this avoidance was actually due to pheromones released by the worms when they were shocked and served as a signal for which direction to turn to avoid punishment [74,75]. To address this potential issue, a group of *H. verbana* were trained using a new chamber for every trial so that there was no chance to utilize a pheromone signal deposited from the preceding trial. Decreases in escape latency and 2 hr retention were still observed, supporting the conclusion that these are learning and memory based behavioral changes and not due to pheromone signaling.

Injury to the posterior sucker produced dramatic changes in *H. verbana* during operant conditioning. Consistent with a previous study from our group [57], injury produces sensitization to non-nociceptive mechanosensory stimuli applied to the injured area over the entire 12-day testing period, comparable to primary allodynia in mammals. Surprisingly, injured animals were not more resistant to crossing the gravel barrier despite this injury-induced mechanosensory sensitization. Instead, the initial (trial 1) escapes latencies in injured animals were considerably lower compared to non-injured leeches tested in the same 40 g gravel condition. In addition, injured *H. verbana* exhibited no evidence of operant conditioning (i.e., no decrease in escape latencies over the five trials) and no evidence of 2 hr retention. The injury-induced decrease in escape latency was confirmed in separate experiments comparing injured-day 0 animals and non-injured leeches in the 40 g condition.

One potential reason for decreased escape latencies in injured-day 0 leeches is that injured animals have increased locomotion. However, time spent crawling was actually less in injured compared to non-injured animals. When the rate of crawling was measured (number of steps/trial), no differences were observed between the injured-day 0 and non-injured animals. Collectively, these results suggest that while crawling was affected by injury, injury-induced decreases in escape latency were unlikely to be due to increased crawling. Interestingly, increased time swimming was observed in injured *H. verbana*. This was surprising because leech swimming usually occurs at a depth of ≥20 mm [77] and the water depth for our experiments was only 10 mm. Swimming was almost entirely absent in non-injured animals and when injured leeches swam they had to tilt onto their sides to compensate for the shallow depth (S3 Video). Time spent swimming was greatest in injured-day 0 leeches and by day 12 swimming time had decreased but was still elevated compared to non-injured leeches. However, the amount of time swimming increased only 2–4 secs per trial, so it is not clear if this enough to directly impact escape latencies.

The effect of injury on negative phototaxis was quite dramatic. In the negative phototaxis assay on injured-day 0, *H. verbana* exhibited substantially reduced escape latencies indicating increased aversion to light. Given the magnitude of this effect, it is proposed that injury induces an increased aversion to light that is the primary driver for reduced escape latencies in injured-day 0 animals. That is, motivation to avoid illuminated areas has increased to promote faster escape despite the gravel obstacle. *H. verbana* detect light via an array of simple eyes distributed all along the body on both the dorsal, ventral and lateral surfaces [72]. These photosensitive cells are all peripheral neurons and are colocalized with low threshold mechanosensitive cells that detect water movement, i.e., waves. Both the photo- and mechanosensitive cells project axons to the CNS and while *H. verbana* do not possess an imaging-forming visual system [72,78,79], there is evidence of visual integration in terms of detecting and orienting to visual stimuli that represent potential prey [71,80,81]. *H. verbana* can detect patterns of light and shadow associated with waves moving across the surface and can integrate this visual information with input from the mechanosensory neurons that detect the mechanical component of waves for the purposes of prey localization [71,76,82].

It is possible that injury-induced changes in the visual system also contributed to changes in exploratory behavior following injury. The overall time dedicated to exploring did not differ between injured and non-injured animals, but non-injured leeches preferentially explored the surrounding chamber while injured leeches showed a preference for exploring the gravel obstacle. The gravel obstacle may be a potent visual stimulus in that it will be a dark region that contrasts strongly with the surrounding illuminated chamber. It is also possible that the height of the 40 g gravel obstacle creates a visual impediment that prevents rapid escape to the dark chamber. From a neural circuit perspective, these photosensors are well-placed to communicate injury-induced sensitization to the rest of the CNS. *H. verbana* photosensitive cells have input to the S interneuron network, a linear array of electrically-coupled neurons that extends the length of the animals [83,84]. S-cells receive input from both photo- and mechanosensory neurons [35,38,65,85,86] and have outputs to motor neurons and serotonergic cells [87,88]. Functionally, the S-cell network is required for sensitization of the whole-body shortening reflex but may also have an arousal-like function that contributes to the earliest stages of behavioral choice [46,89–91].

Injury-induced changes in behavior are thought to have evolutionary adaptive value by preventing or minimizing the likelihood of future damage or even death [2,4,5,92]. We propose that the observed injury-induced behavioral changes leading to reduced escape latency represent an enhanced predator avoidance response in *H. verbana*, comparable to injury-induced increases in predator avoidance observed in squid, *Drosophila*, and amphipod crustacea [5,92–94]. Fresh water leeches are primarily preyed upon by fish [95] which undoubtedly produce waves as they approach. As already stated, leeches can detect both visual and mechanical stimuli produced by waves associated with prey movement, but it is unknown how they would distinguish predators *versus* prey. Therefore, an effective predator avoidance response for an injured leech may be to pre-emptively find shelter in a dark, hidden space (e.g., under a rock) until the injury has sufficiently healed. Increased negative phototaxis, changes in exploratory behavior, and perhaps changes in swimming, may all contribute in accomplishing this goal. Increased predator avoidance may also influence other observed behavioral changes. The shift in exploratory behavior towards the gravel obstacle may represent an attempt to find a substrate to hide under. Increased swimming may benefit the leech in that it now has potential escape routes in three dimensions as opposed to more limited options when the leech is crawling on a substrate (although our placing the leeches in relatively shallow chambers likely interferes with swimming). As the animal recovers from injury negative phototaxis decreased and initial escape latency increased, suggesting that the need for enhanced predator avoidance has passed. Note that sensitization of withdrawal response to non-nociceptive mechanosensory stimuli did not recover during this period, consistent with prior observations [57]. This illustrates differences in the effects of injury on reflexive responses compared to more complex cognitive and motivational behaviors associated with the operant learning task.

The effects of injury on learning and memory were complex and varied over the post-injury period. On day 0, there was no evidence of operant learning or memory and this coincided with increased negative phototaxis and enhanced escape from the illuminated chamber without learning. We propose that this reflects an effect of cognitive bias where the injured state imposes shifts in perceptual and motivational processes that emphasize heightened or more direct responses to environmental stimuli and behaviors that more quickly satisfy immediate drives, e.g., increasing vigilance to avoid further injury as observed in mammals [96,97]. Here the increased negative phototaxis reflects the perceptual change and the more rapid escape from the illuminated chamber represents the increased motivational drive. This cognitive bias towards increased vigilance comes at the expense of the operant conditioning by monopolizing attentional resources, leading to a failure to recognize or attend to other environmental stimuli [98,99]. Put another way, the enhanced visually driven escape response may compromise attention to other environmental stimuli, e.g., the gravel obstacle in the connecting chamber. On day 6 sufficient recovery from injury has occurred to permit a shift in cognitive bias back to pre-injury levels that can support operant conditioning, although 2 hr retention is still disrupted. By day 12, both operant conditioning during trials 1–5 and 2 hr retention had returned. The differences between post-injury day 6 and 12 may reflect differential recovery of two distinct memory systems, a short-term, working memory-like process that operates during the five-trial training phase that has recovered by day 6 and a longer-term memory that corresponds to the 2 hr retention test that does not recover until day 12. Persistent pain has been shown to disrupt memory in both humans and animal models [100,101]. Activity-dependent synaptic plasticity, e.g., long-term potentiation (LTP), are thought to contribute to the cognitive and motivational effects of injury. Alternatively, pain has been shown to disrupt LTP which results in interfering with memory formation [102–106]. Nociceptive stimulation can induce LTP in both *H. verbana* nociceptive and non-nociceptive mechanosensory synapses that exhibit considerable conservation of cellular mechanisms with synaptic plasticity in vertebrates, e.g., the involvement of NMDA receptors, CamKII, PKM, and endocannabinoids [43,49,107,108]. Whether these processes also apply to leech visual synapses is currently unknown.

These findings are relevant to delineating sensory versus possible affective components of pain in *H. verbana* and perhaps other invertebrates. In rodent pain studies, there is increased emphasis to move beyond the traditional tests of reflexes and develop behavioral tests that assess cognitive, motivational, and arousal processes contributing to the affective elements of pain [12,16,17]. Can this be applied to invertebrate studies of pain as well? The idea of invertebrates experiencing the affective component of pain or having emotions at all is controversial [96,109–112]. Part of the problem lies in ascertaining whether persistent behavioral changes presented as evidence of emotion-like processes in invertebrates can be explained by other forms of behavioral modulation. For example, a previous study from our group identified putative evasion behaviors in *H. verbana*, defined as either hiding the injured sucker underneath the body or constantly crawling, both of which were enhanced following injury [64]. These findings can be interpreted as evidence that the injured leech was pre-emptively avoiding further mechanosensory stimuli and represents an affective-like state. A valid counter argument, however, is that the observed increases in evasion behavior were simply sensitization of withdrawal reflexes and locomotory behaviors.

One way to address this issue is to utilize Ledoux’s (2012) characterization of emotions in non-human animals as being mediated by “survival circuits”, separate sensory, motor, and integrative elements that are coordinated in a way to provide an adaptive response to changes in internal/external conditions. Survival circuits mediate changes in arousal, changes in motivation and reinforcement relevant to learning and memory, and how innate and external stimuli initiate specific behaviors, combining to produce the features of emotional behavior [112]. Survival circuits are not limited to vertebrates [112] and examples include changes in predator/prey behaviors in cephalopods [5,113] and context-dependent changes in responses to visual stimuli in *Drosophila* [114]. In the current study, injury in *H. verbana* induced increased negative phototaxis (changes in motivation and responsiveness to external stimuli), altered exploratory behavior (changes in attentional bias and initiation of innate behaviors), disrupted learning and memory processes (changes in cognitive bias and responses to external conditions), and altered in locomotion (changes in arousal and initiation of innate behaviors). Individually, these changes may not necessarily constitute an affective behavior, but they do meet criteria as potential survival circuits and are expressed in a combined, coordinated manner to constitute evidence of an affective-like state following injury. This is not to say that leeches experience, for example, pain-induced fear that is equivalent to fear in humans or even rodents. Rather, it may be more accurate to say that *H. verbana* and other invertebrates exhibit a capacity for affective behaviors that are part of an adaptive response to pain that utilizes comparable mechanisms as described in mammals as emotions but is not as complex (although this too may vary between species). This is analogous to studies of learning and memory. In general, invertebrates do not have the same cognitive capacity most vertebrates (with some notable exceptions, e.g., cephalopods and honey bees), but no one doubts that invertebrates are capable of learning and memory and there is certainly evidence of shared cellular mechanisms across vertebrate and invertebrate phyla [115,116]. Consequently, these findings in *H. verbana* warrant further examination of the physiological and cellular processes mechanisms of these putative survival circuits, e.g., studying the roles of serotonin, dopamine or endocannabinoids in mediating changes in photosensory signaling to the CNS or changes in the crawling and swimming circuits, thereby providing a mechanistic basis for studying potential shared processes mediating these putative survival circuits.

## Supporting information

S1 Fig Effects of increasing amounts of gravel on escape latency and aversiveness of gravel. (A) Escape latency during trial 1 was higher when greater amounts of gravel were in the connecting chamber. Over five trials, latency significantly decreased in the 20 and 40 g groups. *p < 0.05 refers to effect of trial and ****p < 0.0001 refers to effect of group. (B) Leeches exhibited significantly more withdrawal responses when making contact with 40 g of gravel compared to the minimum amount of gravel (10 g). **** p < 0.0001 indicates post-hoc pairwise difference between the 40 and 10 g groups.
(TIF)

S2 Fig Retention (memory) of operant conditioning. Following 8 training trials at 5 min ITI, retention of the reduced escape latency was observed 1 hr following training, but not after 24 hrs.
(TIF)

S1 Video Example of short escape latency during training, i.e., animals that did escape well within 180 sec trial duration. (MP4)

S2 Video Example of withdrawal response from the 40 g gravel obstacle. (MP4)

S3 Video Example of swimming behavior in the arena. Because of the shallow depth, leeches often tilted on their sides to be able to complete the S-shape undulations required for swimming. (MP4)

S4 Video Example of *H. verbana* exploring the open regions of the illuminated chamber. The recording ends with the leech crossing over the gravel barrier to reach the dark chamber. (MP4)

S5 Video Example of *H. verbana* exploring the gravel barrier. (MP4)
