# Supplementary figures and images for: Injury alters sensory, motor, and integrative elements underlying operant conditioning in the medicinal leech
Source: PLoS One. 2025 Jun 12;20(6):e0326039. doi: 10.1371/journal.pone.0326039 (PMC12161587; doi:10.1371/journal.pone.0326039)

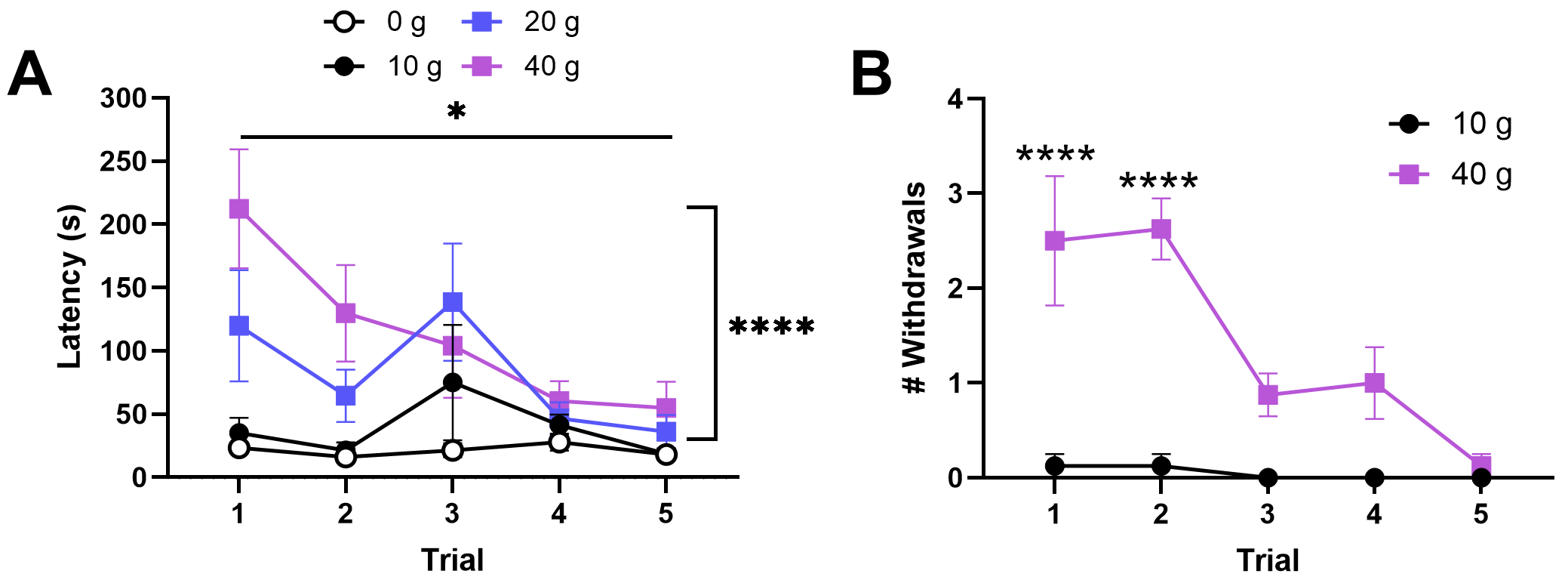

Supplement: S1 Fig — (A) Escape latency during trial 1 was higher when greater amounts of gravel were in the connecting chamber. Over five trials, latency significantly decreased in the 20 and 40 g groups. *p < 0.05 refers to effect of trial and ****p < 0.0001 refers to effect of group. (B) Leeches exhibited significantly more withdrawal responses when making contact with 40 g of gravel compared to the minimum amount of gravel (10 g). **** p < 0.0001 indicates post-hoc pairwise difference between the 40 and 10 g groups. (TIF) [file pone.0326039.s001.tif]

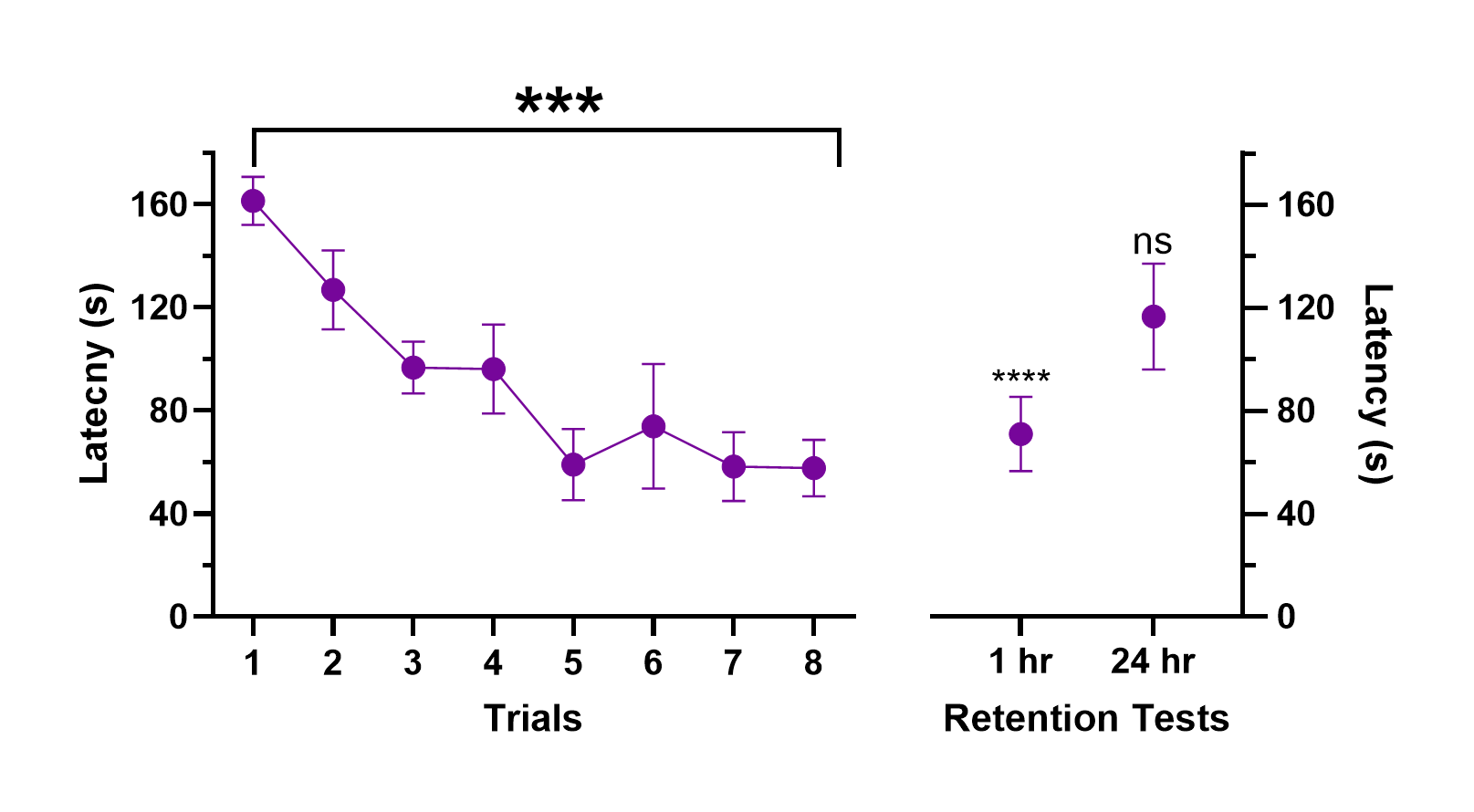

Supplement: S2 Fig — Following 8 training trials at 5 min ITI, retention of the reduced escape latency was observed 1 hr following training, but not after 24 hrs. (TIF) [file pone.0326039.s002.tif]
